# Editorial: The latest advances in transplantation for AML

**DOI:** 10.3389/fonc.2023.1189554

**Published:** 2023-04-05

**Authors:** Patrizia Chiusolo, Fabio Ciceri, Simona Sica

**Affiliations:** ^1^ Dipartimento di Diagnostica per Immagini, Radioterapia Oncologica ed Ematologia, Fondazione Policlinico Universitario A. Gemelli IRCCS, Roma, Italy; ^2^ Sezione di Ematologia, Dipartimento di Scienze Radiologiche ed Ematologiche, Università Cattolica del Sacro Cuore, Roma, Italy; ^3^ IRCCS Ospedale San Raffaele, University Vita-Salute San Raffaele, Milan, Italy

**Keywords:** AML, relapse, stem cell transplant (SCT), genomic, DLI

Allogeneic stem cell transplantation (alloSCT) is the optimal treatment strategy for adult acute leukemia patients due to its ability to reduce the risk of relapse. The ELN group recommends the consideration of alloSCT in fit adults with AML in CR1 who have a predicted relapse risk of 35 to 40% and a suitable donor (1). Risk stratification is based on clinical factors, such as age and gender, as well as cytogenetic risk based on karyotyping results and more recently on Measurable Residual Disease (MRD) assessment during treatment and mutations of prognostic significance in genes like FLT3, NPM1, ASXL1, RUNX1, and TP53 as described in the 2022 ELN classification.

Despite these considerations, disease relapse represents the major cause of treatment failure, so a better stratification of patients based on biological and molecular characteristics, giving the opportunity to optimize conditioning regimen and administrate chemo-immunotherapy after alloSCT, would prevent relapse.

An increasing number of genetic and epigenetic abnormalities have been shown to display the prognostic value in acute myeloid leukemia (AML); in this context, in a monocentric experience, (2) focused their attention on the prognostic impact of the mutational pattern of AML on relapse and survival after alloSCT. They found that FLT3, TP53, and WT1 mutations were predictive variables associated with a higher rate of relapse occurrence after transplant. In a retrospective analysis of 96 AML patients, they found that TP53 mutations conferred a four-times risk of relapse. In a Cox regression model for DFS, they found that not only TP53, WT1, and FLT3 but also NRAS mutations were associated with a reduced outcome.

Baseline genomic features seem to have an important role in predicting survival regardless of the age of AML patients. On the same topic, (3) in a single-center retrospective study determined the impact of targeted therapy vs intensive chemotherapy on the outcome of AML patients aged 60 to 75, demonstrating that the baseline genomic features together with the transplant procedure are the most important independent predictors of overall survival (OS) in the subset of patients aged 60 to 75.

In this context, the intensification of the conditioning regimen may be able to improve the prognosis in terms of better survival. In a retrospective matched pair study, (4) analyzed the role of the combination of decitabine (Dec) and mBuCy for intermediate- and high-risk AML patients undergoing allogeneic stem cell transplantation from a sibling or haploidentical donors, compared to mBuCy alone. The choice of Dec was based on the antileukemic efficacy of the drug, its synergistic action with Bu, and its favorable immunological effects. The study cohort consisted of 156 patients of whom 58 received decitabine followed by mBuCy. The study demonstrated a reduced incidence of acute GvHD grade II-IV (15.2% vs 32.6%, p=0.033) but a higher rate of cGVHD. From the point of view of transplant outcome, the intensified regimen with decitabine was associated with better overall survival (OS) (81% vs 59.4%, p = 0.03). This was confirmed in the haploidentical transplant subgroup (84.8% vs 58.2%, p=0.047), and the cumulative incidence of relapse (CIR) was significantly lower in the Dec-mBuCy group (17.9% vs 40.0%, p=0.036). Beyond the limitation of a retrospective monocentric study, Dec-intensified mBuCy can be considered an opportunity for patients with intermediate- and high-risk AML.

(5) focused their review on cellular and biological-based therapy after alloSCT, underlying that the possibility of success of transplant is related to the capacity to dissect GvHD from the GvL effect, which is related to the ability to trigger a specific donor T-cell mediated immunologic reaction. In this context, DLI represents one of the most used strategies with the purpose of intensifying GvL, but recently it has been associated to target therapies and novel molecules in order to test their efficacy and safety after transplant. In this setting, the association between DLI, Azacitidine (AZA), and Venetoclax in 26 AML patients who relapsed after transplant has been recently published with an overall response rate of 61.5% (voce 98 di Bonifazi diventa (6).

(7) focused their attention on the use of AZA and DLI in their meta-analysis. Searching in PUBMED, EMBASE, and Cochrane Central Register of Controlled Trials, they found and analyzed 13 studies involving 811 patients. With this approach, the rate of cumulative complete remission and partial remission and 2-year OS seems to be 30% and 31%, respectively. From this meta-analysis, several points of discussion emerged regarding the effectiveness of this therapeutic approach. The factors associated with a worse outcome seem to be being over 55 years of age, a blast count at recurrence greater than 20%, the presence of unfavorable cytogenetic alterations, and finally an early post-transplant recurrence (< 6 months).

In summary, the collection of articles on this Research Topic provides an update on the progress in the understanding of both biological characterizations of AML and therapeutical approaches before and after transplant in order to reduce relapse. Molecular stratification has become fundamental not only for risk stratification but also for the identification of druggable mutations that could be used not only in the phase preceding transplant in order to reduce minimal residual disease but also in the post-transplant period as a further consolidation of the remission obtained. From the review of (5) and the meta-analysis from (7), we can assume that there is a need to find effective regimens after transplant that are able to combine the donor T-cell immunological effect with target therapies or novel molecules, and for this specific aim, we need to deepen the biological insights underlying post-transplant relapse.

## Author contributions

PC wrote the Editorial, FC and SS approved it. All authors contributed to the article and approved the submitted version.
